# Pan-Transcriptome Analyses of Multiple Tissues and Growth Stages Create Expression Atlases for the Silkworm *Bombyx mori*

**DOI:** 10.3390/ani16071046

**Published:** 2026-03-29

**Authors:** Linrong Wan, Yaming Jiang, Cheng Zhang, Mengyao Lu, Aijun Ye, Jiezhi Yang, Cao Deng, Yi Wang, Wenfu Xiao

**Affiliations:** 1 Sericultural Research Institute, Sichuan Academy of Agricultural Sciences (SAAS), Nanchong 637000, China; 2 Experimental Center, Neijiang Academy of Agricultural Sciences, Neijiang 641000, China; 3 Department of Bioinformatics, DNA Stories Bioinformatics Center, Chengdu 610000, China; 4 Department of Sericulture and Edible Fungi, Yibin Academy of Agricultural Sciences, Yibin 644000, China

**Keywords:** *Bombyx mori*, pan-transcriptome, RNA atlas, tissue-specific gene, house-keeping gene, alternative splicing

## Abstract

Transcriptome atlases can be used to examine the spatiotemporal dynamics of gene expression, thereby enabling the generation of genome-wide resources for understanding complex biological processes. In the silkworm *Bombyx mori*, transcriptomes serve as crucial datasets for elucidating the mechanisms underlying economically important traits. In this study, we integrated 832 transcriptome datasets across all developmental stages and tissues and performed whole-genome-scale transcriptome sequencing (RNAseq) on five critical tissues from silkworm strains Xian8 and 9211. We identified 5773 and 3323 housekeeping genes expressed across all developmental stages and tissues, respectively, and these genes were primarily enriched in cellular signaling, transport, structural organization, DNA repair, and RNA processing pathways. We also identified 27 stage-specific genes and 58 tissue-specific genes, providing candidate markers for future single-cell and spatial transcriptomics. A large number of alternative splicing events were detected from 832 NGS samples, indicating the critical roles of alternative splicing in silkworm development. Interestingly, only 10 long-read full-length transcriptome samples from Xian8 and 9211 yielded results comparable to the NGS in terms of novel genes and alternative splicing events, and these multi-tissue comparative analyses also revealed significant differences in alternative splicing patterns, underlining the necessity of long-read sequencing for such research. These datasets not only advance functional genomics research in Lepidoptera but also provide molecular signatures for silkworm strain-specific comparisons and association analyses with differential phenotypes. Silkworm pan-transcriptomics, by analyzing multidimensional transcriptional regulatory networks and gene-expression dynamics, can facilitate multidisciplinary integration and accelerate the breeding of high-yield and high-quality silkworm varieties.

## Simple Summary

The silkworm (*Bombyx mori*) is a key model for lepidopteran research and a core economic insect for global sericulture. Here, we constructed a comprehensive silkworm pan-transcriptome resource: an integrated dataset covering the species’ full transcriptomic landscape across diverse tissues, developmental stages, and strains. We combined 832 samples (888 next-generation sequencing transcriptomes) spanning the full silkworm life cycle and all major tissues, with additional whole-genome and long-read full-length transcriptome data from five key tissues of Xian8 (限8) and 9211, two widely used commercial silkworm strains in China. Our key findings include a high-confidence panel of housekeeping genes and tissue/developmental stage-specific marker genes for silkworm transcriptomic research, a genome-wide map of alternative splicing events revealing the critical role of post-transcriptional regulation in silkworm development, and validation of the irreplaceable value of long-read sequencing for detecting novel genes and splicing variants. All data from this study are freely available via our interactive EXP832 web platform and the NCBI public database. This resource provides a foundational reference for lepidopteran functional genomics research and supports molecular breeding of high-yield, high-quality silkworm varieties.

## 1. Introduction

Pan-transcriptome refers to a set of transcriptomic data that are used to map the distribution and expression levels of RNA transcripts across different tissues, conditions, or developmental stages [1]. These data provide a comprehensive view of the expression patterns of the entire genome that can be used to continuously revise the functional annotations of the genome and to understand the molecular mechanisms of complex life processes [2,3,4]. Transcriptomes are powerful tools with which to identify tissue-specific gene (TSG) expression, understand developmental processes, explore disease mechanisms, discover regulatory relationships, and facilitate comparative studies [5,6,7].

Transcriptome atlases typically arise from projects that utilize high-throughput sequencing techniques such as RNA-Seq. These technologies can produce the extensive datasets required to assemble a nuanced representation of the transcriptome. In humans, the Genotypic-Tissue Expression Alliance (GTEx) and the International Human Epigenomic Consortium (IHEC) have amassed numerous datasets. These have not only identified the quantitative genetic traits that influence gene expression but have also pinpointed those that exhibit tissue-specific patterns. This has led to a deeper understanding of the epigenetic control mechanisms underlying cell states associated with both human health and disease [8,9]. In animal genomics, the examination of expression profiles across various tissues in pigs [10], cows [11], and chickens [12,13] has been instrumental in refining genome assembly and annotation. Such advancements are pivotal for gaining a deeper understanding of mammalian evolution [14,15]. In other domains, individual transcriptome databases have been developed for a range of plant species, including alfalfa (Medicago truncatula) [16], soybeans (Glycine max) [17], barley (Hordeum vulgare) [18], and yeast (Saccharomyces cerevisiae) [19]. These resources are beneficial for agricultural breeding endeavors. In entomology, transcriptomics has predominantly been harnessed to explore areas such as developmental biology, evolutionary processes, and immune system studies [20,21].

The silkworm *Bombyx mori* is the only economically important domesticated insect species. *B. mori* has played an important role in the development of human civilization [22,23]. The silkworm *Bombyx mori* is not only a pillar economic insect for global sericulture, but also a classic lepidopteran model organism widely used in applied research fields including materials science (e.g., development of silk-based functional biomaterials [24]) and medicine (e.g., as a bioreactor for recombinant pharmaceutical protein production [25]), as well as a well-established research subject for methodological innovation in molecular biology [26,27] and bioinformatics [28]. As a model insect of Lepidoptera, *B. mori* is involved in the study and control of insect pests [29]. Transcriptome research using silkworms has achieved important results. Chen et al. studied the silk glands of silkworms using PacBio single-to-long-reading sequencing and contributed a large amount of transcriptome data [30], and Ma et al. conducted in-depth research through single-cell sequencing of the silk gland, which led to the identification of genes and cells related to silk yield [31]. However, to date, no studies have produced a comprehensive RNA atlas encompassing multiple tissues, developmental stages, and silkworm strains.

In this study, an RNA atlas for the silkworm was produced using genome data, 832 manually verified transcriptome datasets, and 10 full-length RNAseq datasets of the strains Xian8 and 9211. Tissue- and development-specific genes and housekeeping genes (HKGs) in silkworms were analyzed, with particular attention paid to variable splicing of HKGs. Our results were corroborated by full-length RNAseq. We identified 27 development-stage-specific genes and 5773 HKGs consistently expressed throughout development, along with 58 TSGs and 3323 ubiquitously expressed HKGs across all tissues. Using these data, we conducted a comprehensive analysis of alternative splicing patterns and their temporal dynamics. Notably, third-generation sequencing (TGS) detected 11,899 novel alternative splicing events across 4219 genes in just 10 samples, far exceeding the number identified in 832 second-generation sequencing samples. The results highlight TGS’ superior ability to uncover alternative splicing diversity. The value of this study lies in its analysis of the silkworm RNA atlas and the sharing of full-length transcript information from different tissues of two commercial silkworm strains, thereby providing theoretical support for subsequent silkworm research and a reference for studying RNA atlases in other species.

## 2. Materials and Methods

### 2.1. Collection of Silkworm Transcriptome Datasets

**Figure 1:**
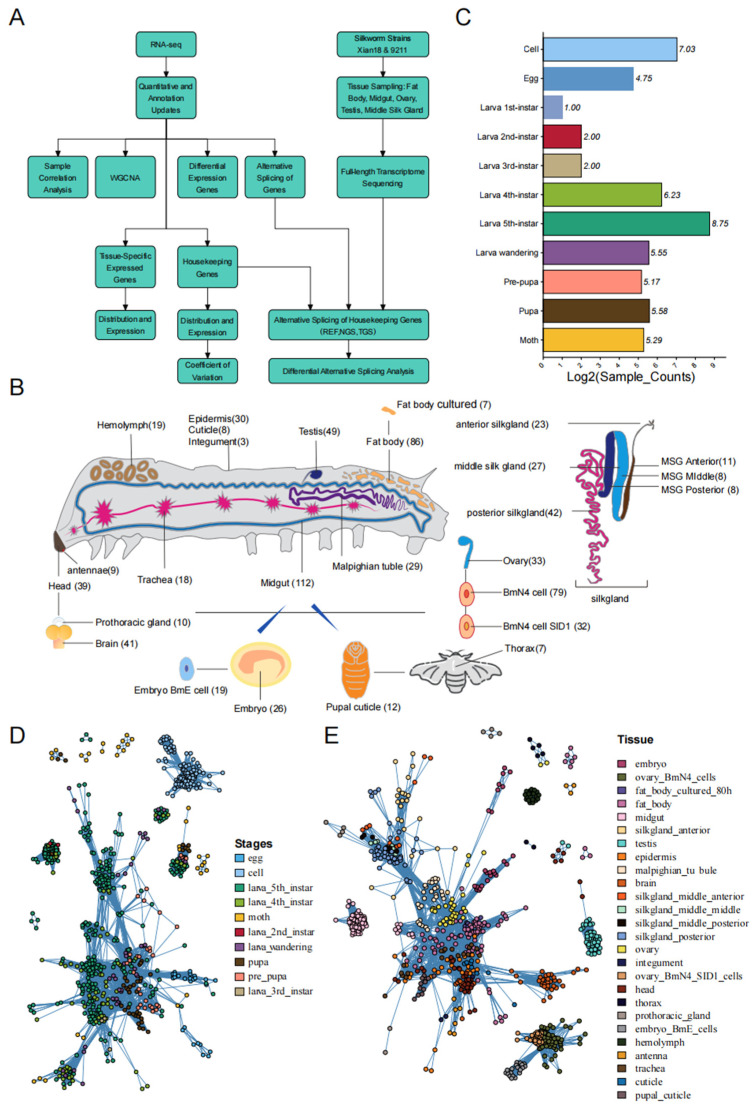
The Analysis Pipeline And The Distribution Of Samples. (**A**) Analysis pipeline and sample distribution. (**B**) Sample distribution by tissue. and (**C**) Growth stages. Cluster analysis based on samples from different developmental stages (**D**) and tissues (**E**). Each node represents a tissue/developmental stage. The sizes of the nodes are fixed. Node colors indicate different tissues.

Genome and gene annotation of *B. mori* were downloaded from SilkBase (http://silkbase.ab.a.u-tokyo.ac.jp, accessed on 1 July 2025) [32]. The silkworm transcriptome data were downloaded from the National Center for Biotechnology Information (NCBI) website (https://ncbi.nlm.nih.gov/), comprising a total of 832 entries, and the information registered in the transcriptome has been manually verified [33]. Figure 1 presents specific details of the transcriptome registration information, as well as the age and tissue distributions.

### 2.2. Transcriptome Alignment and Annotation Updating

The collected transcriptome data included both single-end and paired-end sequencing types; these were quality-controlled separately using Fastp (version 1.3.0) [34]. Following this, an index was created, and alignment was carried out using Hisat2 (version 2.2.2) [35]. The alignment results were then sorted using Samtools (version 1.23.1) [36]. Samples with multiple sequencing runs were consolidated using Samtools with the parameters (merge -urc -f -b).

The transcriptome data were assembled for each sample using StringTie (version 2.2.1) [37] with default parameters. Subsequently, all sample gtf files were merged using StringTie with the parameters --merge and -G reference.gff3. PASA is a tool for eukaryotic genome annotation that automates gene structure modeling from transcriptome data, aligns the results with experimental data, and updates gene annotations, including splicing variants [38,39,40]. The annotation file was updated using PASA version 2.5.2. After updating, the gff3 file was converted to gtf format using gffread [41] version 0.12.1 with the parameter -T. Finally, alternative splicing events were analyzed using SUPPA [42] version 2.3 with the following parameters: suppa.py generateEvents -f ioe -e SE SS MX RI FL.

### 2.3. Gene Expression

StringTie [37] (version 2.2.1, -B option) was used for the quantification of each sample. Subsequently, the quantification results were aggregated across all samples using the software’s integrated prepDE.py script, along with a tailored script. Batch effect correction was performed on the gene expression count matrix using the ComBat_seq function implemented in the R package sva [43] (v3.52.0). This process culminated in the generation of a comprehensive gene expression matrix.

### 2.4. Sample Correlation Analysis

Inter-sample correlation analysis is a key statistical method for assessing the similarity or degree of association in gene expression between different samples. The analysis can help researchers understand biological differences and check data consistency among samples.

We constructed a correlation network diagram to analyze and illustrate relationships among samples as a visual representation of the degree of similarity or correlation.

Correlation analysis of samples was performed using the corr.test function from the psych package (version 2.2.3) [44] in R (4.3.1). Missing values were handled by pairwise deletion, Spearman’s rank correlation coefficient was calculated, the Holm method was applied to correct *p*-values for multiple comparisons, and the significance level was set at 0.05. The network was constructed using the igraph [45] package in R.

### 2.5. Coefficient of Variation Analysis

The coefficient of variation (CV, or dispersion coefficient) is a statistical metric quantifying data variability relative to the mean. For each gene, CV was calculated as the ratio of the standard deviation to the mean of its expression across samples, with higher CV indicating greater expression instability.

We first calculated the CV for all genes. The genes were then categorized into three groups based on quartiles of the CV distribution: high variation (upper 25%), medium variation (25–75%), and low variation (lower 25%). The housekeeping genes (HKGs) were subsequently mapped to the corresponding classification system. For Low CV group, we divided these HKGs into three subgroup according to their expression level: TPM: ≥50, TPM: 10–50, TPM: 1–10. Next, we identified and counted the number of HKGs within each group.

### 2.6. Identification of Tissue-Specific and HKGs

The tau expression index, counts, and the Gini index are metrics used to assess gene expression levels in specific tissues. The higher the values of these three indices, the more specific the gene expression is to the target tissue; conversely, lower values indicate more conserved expression. We used tspex [46] (version 0.6.1) to calculate tau (with default parameters), counts (--threshold 1), and the Gini index (with default parameters) values for subsequent analysis of tissue-specific and HKGs.

Tissue-specific genes, also known as luxury genes, are selectively expressed in different cell types. The products of these genes confer specific morphological and structural characteristics, as well as unique physiological functions, on various cell types. We set a threshold of 0.8 to identify TSGs, meaning that a gene needed to have a value greater than 0.8 in at least two of the three metrics. Furthermore, the gene needed to pass the Venn test to be recognized as a tissue-specific gene for the corresponding tissue.

HKGs are a class of genes that are stably expressed in all cell types, with their products essential for maintaining basic cellular functions. These genes are expressed in all cells, as opposed to “luxury genes” that are expressed in specific cell types. We selected 0.2 as the threshold for screening HKGs. If a gene had values below 0.2 in at least two of the three metrics, we consider it as an HKG.

### 2.7. Weighted Gene Co-Expression Network Analysis

Before analysis, we filtered the selected gene sets, excluding low-quality genes or samples that could introduce instability and adversely affect the outcomes. This rigorous pre-processing step is crucial for enhancing the precision of our subsequent analysis. Initially, the dataset comprised 14,446 genes; after filtering, 14,400 genes were deemed suitable for inclusion in the analysis.

We then utilized the R environment with the R-WGCNA [47] (Weighted Gene Co-expression Network Analysis) package for the analysis. Within this framework, we set specific parameters to optimize network construction: the soft-threshold power was set to 9; the minimum network connectivity (deepSplit) was set to 2, and the network type was set to signed.

GO enrichment: This stage employed a custom script to perform GO enrichment analysis on the TSGs obtained above. The condition for GO enrichment significance was a *p*-value ≤ 0.05.

KEGG enrichment: A custom script was used to perform KEGG enrichment analysis for each TSG set. The KEGG enrichment criterion was *p* ≤ 0.05. The netplot primarily employs the aPEAR package [48] in R (version 4.1.3).

### 2.8. Identification of Gene Alternative Splicing Types

Alternative splicing analysis was conducted utilizing RNA-seq data and analyzed with the rMATs software [49]. Alternative splicing events can be classified into five distinct categories: skipped exons (SEs), mutually exclusive exons (MXEs), alternative 5′ splice site (A5SS), alternative 3′ splice site (A3SS), and retained introns (RIs). rMATs facilitates both quantitative and differential analyses of alternative splicing events, with a specific parameter setting for paired 150 bp reads (-t paired -readLength 150).

### 2.9. Quantitative and Differential Analysis of Gene Alternative Splicing Events

We used rMATs (version 4.2.0) for quantitative and differential analyses of alternative splicing (AS) events with the parameters -t paired --readLength 150; in addition, we employed rmats2sashimiplot (version 3.0.0) for visualization of differential AS events using default parameters. Each AS event corresponds to two isoforms, namely the exon inclusion isoform and the exon skipping isoform. The expression levels of the two isoforms were measured and divided by their effective lengths to obtain the corrected expression levels. Then, the ratio of the total expression of exon inclusion isoforms was calculated and expressed as IncLevel1 (treatment group) and IncLevel2 (control group). Finally, the difference was analyzed, and an FDR ≤ 0.05 was used to identify genes with a significant splicing event (the FDR was defined as the *p*-value after Benjamini–Hochberg correction).

### 2.10. Silkworm Strains, Tissue Collection, and RNA Extraction

Xian8 (限8) and 9211 are silkworm strains bred by Sericultural Research Institute of SAAS. The Xian8 variety from Japan is a sex-limited silkworm strain, in which female and male individuals can be directly distinguished by their different body color and markings; It also exhibits significant advantages in economic traits compared with laboratory strains such as Dazao. The 9211 variety, originating from China, is known for its exceptional ability to produce high-grade silk cocoons and its strong environmental adaptability. These two silkworm strains were cultured on a diet of fresh mulberry leaves in a carefully controlled environment that maintained a constant temperature of 25 ± 1 °C and adhered to a 12/12 h alternating light/dark cycle.

The tissue samples were collected from the Xian8 and 9211 silkworms on the second day of their fifth larval instar. The selected tissues for sampling comprised the fat body, midgut, testes, ovaries, and the middle silk glands. The entire sampling process was conducted under stringent sterile conditions to ensure sample purity. Specifically, the midgut tissue was meticulously cleaned to reduce the interference from mulberry leaf residue, thereby enhancing the quality of the RNA sample.

Total RNA was extracted using a Fast RNAex Kit (BGMG, Chongqing, China). Thereafter, a PrimeScript™ RT reagent Kit with gDNA Eraser (TAKARA, Kusatsu, Japan) was utilized to eliminate any contaminating genomic DNA from the purified RNA. Subsequently, complementary DNA (cDNA) was synthesized via reverse transcription using the 5× PrimeScript™ mix (TAKARA, Kusatsu, Japan).

### 2.11. Identification and Quantification of Full-Length Transcripts

The full-length transcriptome library for the sample was constructed using the QDL-E V1.1 kit (Qitantech, Chengdu, China) according to the manufacturer’s instructions. The TGS platform is the QitanTech QNome-3841hex (Qitantech, Chengdu, China). Nanofilt (ver. 2.8.0) [50] was used for the quality control of sequencing results, employing default parameters. IsoQuant (v3.4.1) [51] is a bioinformatics software designed for the quantification of full-length transcriptomes using a reference. The program employs a minimap for alignment against the reference genome and corrects splicing sites. We employed IsoQuant to analyze full-length transcriptomes (--sqanti_output --check_canonical --report_novel_unspliced true), yielding genomic annotations and quantification results. Subsequently, we appended source annotations to the obtained results to distinguish transcripts derived from RefSeq (NCBI), NGS, and those discovered by QNome. IsoQuant was further utilized to analyze the newly identified alternatively spliced transcripts, encompassing aspects such as transcription start sites and transcript lengths (SQANTI output format [52]).

### 2.12. Statistics of Newly Discovered Genes and Transcripts

First, a density plot was created from all expression values to compare the sensitivity of the two sequencing platforms for genes with low and high abundance. Before plotting, the following pre-processing steps were taken: (1) TPM values greater than 25 were capped at 25, and (2) genes with TPM less than 0.5 were removed. Subsequently, the number of transcripts from different sources was tallied, and an UpSetR plot was generated. We defined a gene or transcript as not expressed when TPM was less than 0.5. It is important to note that some genes may be detected in second-generation sequencing but not expressed (0 < TPM < 0.5); yet, they are expressed in third-generation sequencing. In such cases, we categorized these as transcripts identified by third-generation sequencing.

### 2.13. Statistics of Newly Identified Alternative Splicing Forms

We used SUPPA2 (version 2.3) to detect differential splicing events and determined the percentage of spliced-in (PSI) values. A PSI value close to 1 signifies that most transcripts encompass the exon under consideration, indicative of a frequently occurring exon inclusion in splicing. To identify disparities in the detection of AS between second- and third-generation sequencing technologies, we focused on transcripts that were distinctive to either third-generation or second-generation sequencing. The term ALL encompasses all transcripts, and NGS denotes transcripts specific to second-generation sequencing, as shown in the UpSet plot. Finally, we applied a PSI threshold > 0.6 to filter and quantify AS events in our samples.

## 3. Results

### 3.1. Summary Statistics of Sequencing Datasets

We conducted a detailed analysis of silkworm genes based on 832 public transcriptome datasets [53], but this was not the main focus of the research. Our focus was on the AS of HKGs, an important aspect of RNA landscapes. To verify and further study the AS of HKGs, we employed full-length RNAseq, which is commercially available, and sampled five key silkworm tissues.

The accuracy of the basic data ensured the reliability of the follow-up analysis results. To improve the accuracy of the analysis, we analyzed the distribution of samples by developmental stage and tissue using the 832 RNA-seq dataset and its corresponding corrected information [53] (Figure 1B,C). This dataset comprised 26 tissues, of which the most of transcriptome data were from the integument, epidermis, and midgut, with 112, 86, and 79, respectively, and the least were from the head (3), fatbody (7), and thorax (7). For the developmental stage, the 5th-instar larvae had the most transcripts (429), followed by in vitro-cultured cells (130) and 4th-instar larvae (74). However, the transcriptomes of the 1st (1), 2nd (3), and 3rd (3) instar larvae contained only a small number of genes, in the single digits. Overall, these transcriptome datasets were accurate and comprehensive.

A sample correlation network was utilized to illustrate the clustering relationships among samples. When the data are accurate, clustering should reflect the similarity among samples. The correlation network diagram (Figure 1D,E) indicated that the samples collected at the same developmental stage or that belong to the same tissue generally clustered together. In contrast, sample groups with different attributes did not cluster together. This suggests that our collected transcriptomic data were accurate. In developmental-stage-based transcriptomic clustering, in vitro-cultured cells were significantly separated from other samples and formed distinct clusters. Additionally, egg samples and all larval-stage tissue samples demonstrated relative independence within the clustering architecture. Similar patterns were observed in tissue-type-based clustering: both hemolymph and testis tissues remained independent across all developmental stages, forming tissue-specific clusters.

### 3.2. Identification of HKGs and Tissue-Specific Genes

HKGs are a class of genes that are stably expressed across all developmental stages and in all tissues, and their gene products are essential for maintaining basic life activities [54]. The gene expression matrix, when integrated with sample metadata using the tspex tool, facilitates the computation of several key statistical measures, including Tau (with default parameters), Counts (-threshold 1), and the Gini index. These metrics were used to identify genes with distinct expression patterns and HKGs across the silkworm genome. Finally, TPM ≥ 1 was used to determine gene expression, and an UpSet plot was used to validate the results.

**Figure 2:**
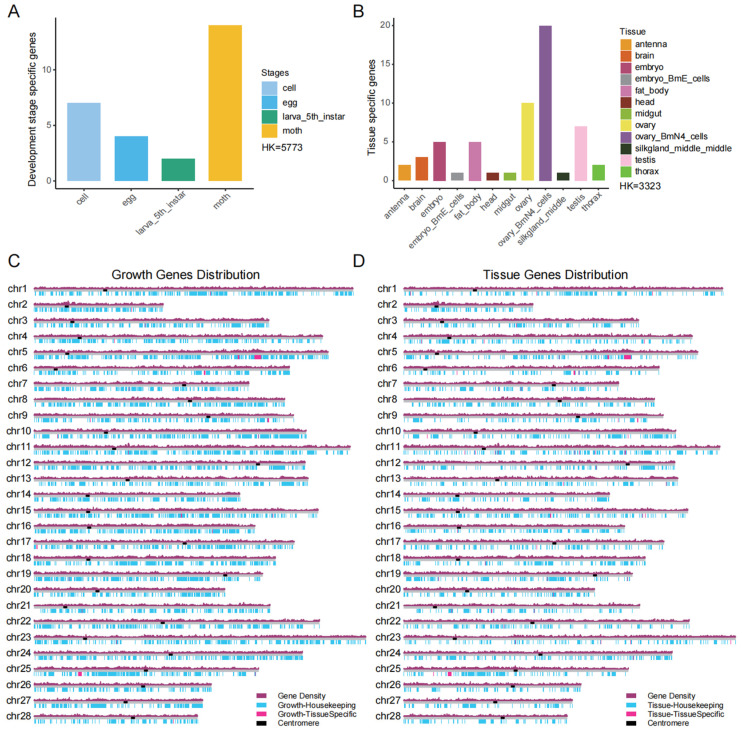
Statistics of housekeeping genes and specific expression genes. (**A**) Housekeeping genes and developmental stage-specific genes were identified with the developmental stage as a reference. (**B**) Housekeeping genes and tissue-specific genes were identified with tissue type as a reference. Growth and development stages or tissues in which no specific genes are expressed are not listed, but they were included in the calculation of housekeeping genes. (**C**) Chromosomal distribution of development-related housekeeping genes and development-specific genes. (**D**) Chromosomal distribution of tissue-related housekeeping genes and tissue-specific genes.

We identified HKGs and stage-specific genes by screening the developmental-stage and silkworm transcript gene-expression data. Our analysis identified a set of genes that exhibited developmental-stage-specific expression across the silkworm life cycle, including cultured cells, eggs, 5th-instar larvae, and adult moths. Additionally, we identified a substantial cohort of 5773 HKGs that demonstrated consistent expression across all examined developmental stages, encompassing even those not explicitly depicted (Figure 2A). In a parallel approach, we conducted a thorough examination of the transcriptome samples, which yielded a set of TSGs along with HKGs. Our analysis revealed TSGs across 10 distinct tissues. Concurrently, we identified a robust set of 3359 HKGs that exhibited ubiquitous expression across all tissues examined (including those not listed; Figure 2B). These genes also exhibited certain distribution patterns: the development-related HKGs were widely distributed across chromosomes 1–28 with uneven density, showing particularly high enrichment on chromosomes 1, 4, and 11, whereas development-specific genes were only present at specific loci on a few chromosomes, including 5, 6, and 25 (Figure 2C). The tissue-related HKGs exhibited a more uniform chromosomal distribution but still showed significant enrichment on chromosomes 11, 15, and 24, whereas TSGs were concentrated at specific loci on chromosomes 5, 6, and 25, with stronger chromosomal preference (Figure 2D).

### 3.3. Functional Analysis and Expression Patterns of HKGs

**Figure 3:**
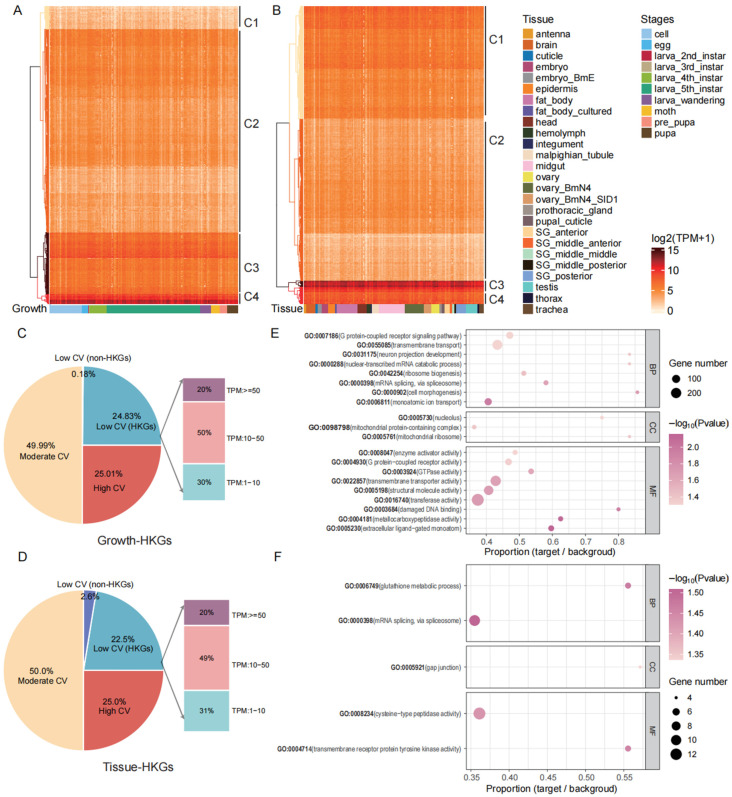
Expression patterns and coefficient of variation in housekeeping genes identified under different reference modes. (**A**) Expression pattern of housekeeping genes in the entire development of the silkworm. (**B**) Expression patterns of housekeeping genes in whole tissues of the silkworm; the horizontal axis represents the samples, while the vertical axis denotes the selected housekeeping genes. The coefficient of variation analysis of housekeeping genes involved in growth and development (**C**) and tissues (**D**). Gene ontology enrichment analysis results of growth-related (**E**) and tissue-related housekeeping genes (**F**).

HKGs of the silkworm should exhibit certain expression characteristics. We constructed heatmaps to verify the results of the HKG analysis and further explore their expression patterns. In the heatmaps categorized by developmental stages (Figure 3A) and tissue types (Figure 3B), we observed that these HKGs were consistently expressed across all developmental stages and in all tissues, supporting our selection of HKGs (Figure 3). Interestingly, we found that all growth-housekeeping genes were assigned to the low coefficient of variation group, while only 0.18% of genes in this Low CV group were non-growth-HKGs. The distribution pattern of tissue-HKGs was highly consistent with this: only 2.6% of genes in the corresponding Low CV group were non-tissue-HKGs. Based on these results, it can be inferred that HKGs as a whole exhibit a significant preference for Low CV distribution compared with the CV levels of all genes across the genome (Figure 3C,D).

GO enrichment analysis of growth-related and tissue-related HKGs (Figure 3E,F and Table S1) revealed that growth-related HKGs were significantly enriched in biological processes including G-protein coupled receptor signaling, transmembrane transport, and neuron projection development, in cellular components including nucleolus and mitochondrial ribosome, and in molecular functions including transferase activity, transmembrane transporter activity, and G-protein coupled receptor activity, with a large number of enriched terms, high gene counts, and high significance levels (–log_10_ (*p*-value) > 1.8), reflecting their core roles in cellular signal transduction, material transport, and basic metabolism; in contrast, tissue-related HKGs were only enriched in biological processes including glutathione metabolism and mRNA splicing, in cellular components involving gap junctions, and in molecular functions such as cysteine-type peptidase activity and transmembrane receptor protein tyrosine kinase activity, with fewer enriched terms, smaller gene counts, and lower significance levels (–log_10_ (*p*-value) < 1.5), suggesting that their functions are more biased toward maintaining tissue-specific homeostasis and metabolic demands.

For the KEGG pathway enrichment analysis (Table S2), Growth_HKG was significantly enriched in non-homologous end-joining (*p* = 0.0316), RNA degradation (*p* = 0.0364), and epithelial cell signaling in Helicobacter pylori infection (*p* = 0.0423), indicating its critical involvement in DNA damage repair, RNA metabolic processing, and cellular response to pathogenic infection. In contrast, Tissue_HKG was enriched in a broader set of pathways, including phagosome (*p* = 0.0109), epithelial cell signaling in H. pylori infection (*p* = 0.0119), RNA degradation (*p* = 0.0151), SNARE interactions in vesicular transport (*p* = 0.0155), sphingolipid signaling pathway (*p* = 0.0332), non-homologous end-joining (*p* = 0.0335), long-term depression (*p* = 0.0434), and mRNA surveillance pathway (*p* = 0.0459), suggesting its multifaceted roles in cellular immune defense, intracellular vesicular trafficking, and neural regulatory functions.

### 3.4. Expression Patterns of Specific Genes

**Figure 4:**
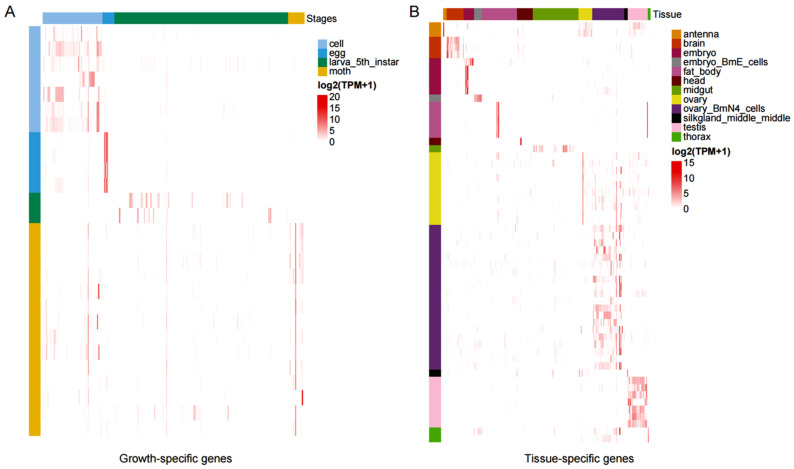
Statistics and expression patterns of specific genes. (**A**) Expression patterns of stage-specific genes expressed during growth and development. (**B**) Expression patterns of tissue-specific genes. In the expression pattern diagram, the horizontal axis represents the samples, and the vertical axis represents the specific genes.

Based on the identified stage-specific and TSGs, we systematically investigated their expression patterns and calculated the CV for each gene. The results are illustrated in Figure 4. Stage-specific genes exhibited predominant expression in their respective developmental stages, whereas they showed significantly lower expression levels or insufficient sample representation in other stages (Figure 4A). The TSGs depicted in Figure 4B maintained high expression levels in their target tissues but showed low abundance or inadequate sample coverage in other tissues. Notably, cell developmental stage-related genes demonstrated the most prominent regulatory characteristics in the stage-specific expression patterns, whereas fat body-specific genes exhibited the strongest tissue exclusivity in tissue-specific expression. In addition to the previously annotated genes, we identified four newly annotated genes among specifically expressed genes: novel_gene_63_65869db7, novel_gene_707_65869db7, novel_gene_679_65869db7, and novel_gene_525_65869db7. Among these, novel_gene_63_65869db7 and novel_gene_679_65869db7 were co-expressed in both tissue-specific and developmental stage-specific categories; novel_gene_707_65869db7 (fat body-specific) was exclusive to tissue-specific expression, and novel_gene_525_65869db7 (moth-specific) was solely within developmental stage-specific genes (Table 1 and Table S3).

### 3.5. Construction and Mining of Regulatory Network

**Figure 5:**
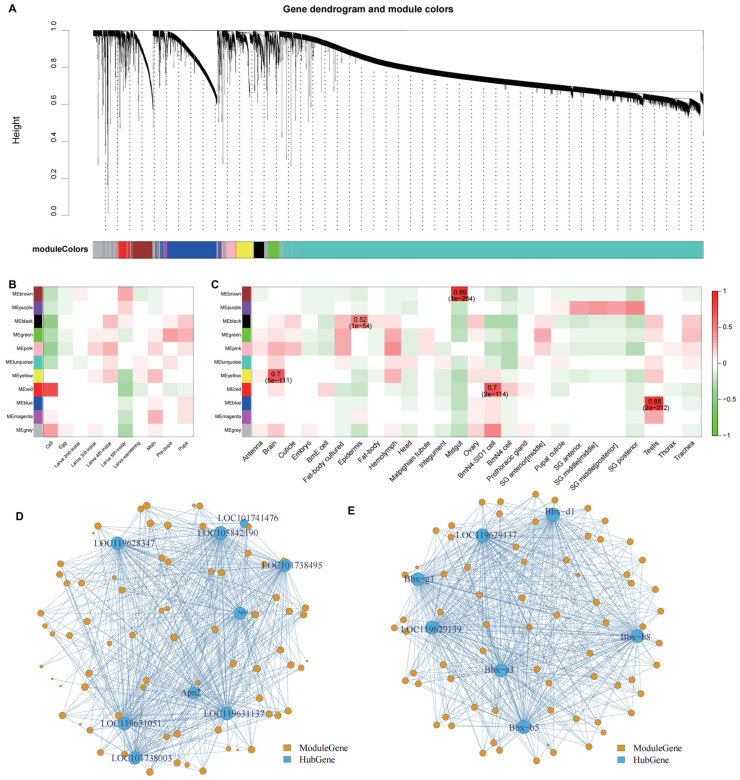
WGCNA of housekeeping genes and specific expression pattern genes. (**A**) The modules and their corresponding genes were classified into gene modules defined by expression patterns. In the module hierarchical clustering tree, different colors denote distinct modules, with gray indicating genes that do not belong to any module. The modules containing type-specific differentially expressed genes are indicated at the bottom. (**B**) Correlation results between gene modules and growth and development stages. (**C**) Correlation results between gene modules and different tissues. The depth of color in the correlation analysis indicates the strength of the correlation. Each row represents a module, and each column represents a phenotype. The color blocks in the diagram represent the correlation coefficients between the modules and phenotypes (Pearson’s correlation), with red indicating positive correlations and green indicating negative correlations. The deeper the color, the more significant the correlation. The numbers within the color blocks are the correlation coefficients, and the numbers in parentheses are the *p*-values. Gene co-expression networks are shown for the brown module (**D**) and the yellow module (**E**). In each network, the yellow nodes represent module genes (modulegene), and blue nodes represent hub genes (hubgene), with node size proportional to the degree of connectivity.

Based on the gene modules identified via WGCNA, the purple module contained the fewest genes (81), whereas the turquoise module had the most (9411), with an average of approximately 1242 genes per module. The genes within each module were sorted by intramodular connectivity, and the top 2% were selected as the hub genes. The results are shown in Figure 5A and Table 2. Subsequently, we explored the correlations between these modules and phenotypes, using both developmental stages and tissue samples as references (Figure 5B,C). In the correlation analysis between developmental stages and gene modules, we identified a significant positive correlation between the red module and the in vitro cultured cell stage of the silkworm, with a correlation coefficient of +0.74 and a *p*-value of 3 × 10^−131^. Additionally, the black and pink modules showed significant negative correlations with the in vitro cultured cell stage, with correlation coefficients of −0.47 and −0.40 and *p*-values of 2 × 10^−43^ and 1 × 10^−30^, respectively (Figure 5C). The correlation analysis between sample tissues and gene modules identified four module-phenotype pairs with correlation coefficients of +0.7 or higher: brown with midgut (+0.89, *p* = 1 × 10^−264^), blue with testis (+0.85, *p* = 2 × 10^−212^), yellow with brain (+0.7, *p* = 5 × 10^−111^), and red with BmN4 cells from the ovary (+0.7, *p* = 2 × 10^−114^) (Figure 5C).

Interestingly, among the nine hub genes identified in the brown module, one gene orthologous to the Drosophila melanogaster salivary gland secretion peptide gene, but functionally unannotated, was the only exception; the remaining eight encoded digestion-related enzymes, including proteases, aminopeptidases, and collagenases (Figure 5D). In the yellow module, seven hub genes were identified, all of which were bombyxin genes of the silkworm; these are commonly referred to as insulin genes in other species (Figure 5E). It is noteworthy that the functions of these hub genes precisely align with those of the highly correlated tissues associated with their respective modules (e.g., the brown module with the midgut, and the yellow module with the brain). Hub genes within the blue and red modules also exhibited a similar pattern (Table S4).

### 3.6. Landscape of Alternative Splicing in Silkworms

**Figure 6:**
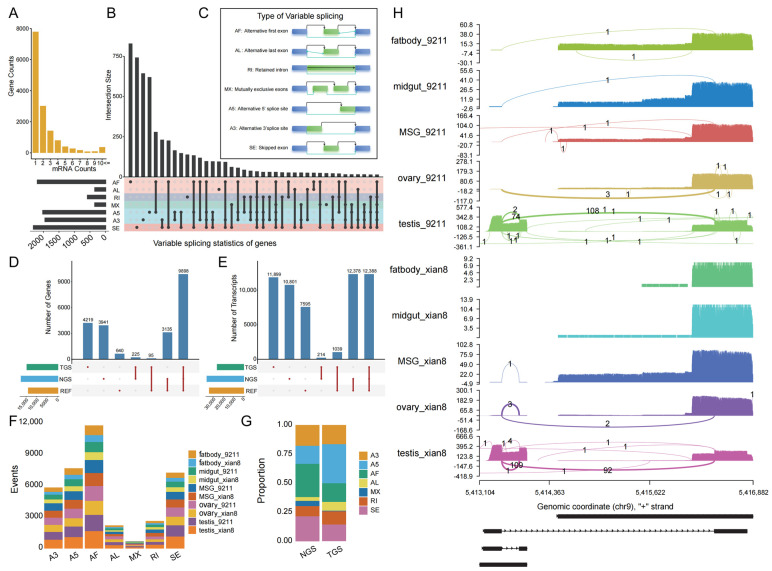
Overview of variable splicing of silkworm genes. (**A**) Distribution of the number of genes with different forms of alternative splicing. (**B**) Statistics of genes with different alternative splicing types; the dots represent the number of genes with this type of variable splicing form, and the connected dots represent the number of genes that intersect with multiple forms of variable splicing simultaneously; the left part illustrates the number of genes with the specific alternative splicing form. (**C**) Schematic diagram of different alternative splicing types; the black lines represent the reference splicing forms of the gene, while the blue lines represent the annotated variable splicing forms. (**D**) Gene-level statistical analysis of alternative splicing events in five different tissues from TGS, NGS, and REF. (**E**) Transcript-level statistical analysis of alternative splicing events in five different tissues from TGS, NGS, and REF. (**F**) Total alternative splicing events identified through TGS of 9211 and Xian8. (**G**) Proportion of novel splicing forms specifically discovered by the TGS or NGS platform. (**H**) Alternative splicing patterns of U1 small nuclear ribonucleoprotein c (U1 snRNP c); the left vertical axis indicates transcripts per million (TPM) expression levels, with the horizontal axis representing genomic transcript coordinates. Numerical annotations denote the sequencing read counts corresponding to each splicing variant.

In the silkworm genome, the distribution of AS events (Figure 6A) demonstrated a negative correlation between the number of such events and the number of genes they affected. Specifically, an increase in the frequency of AS events was accompanied by a corresponding decrease in the number of genes exhibiting this characteristic. The distribution of these splicing forms in the silkworm genome is shown in Figure 6B. There were seven distinct forms (Figure 6C): AF (alternative 5′ splice sites), AL (alternative last exon), RI (intron retention), MX (mutually exclusive exons), A5 (selective splicing at the 5′ end of exons), A3 (selective splicing at the 3′ end of exons), and SE (exon skipping).

In the silkworm genome, the SE AS variant was the most abundant, with a total of 2317 cases. Among AS types, AF ranked second with 2190 cases, followed by A5 with 2015 and A3 with 1943. In contrast, RI had 572 cases, AL had 335, and MX had 336. Among genes with only a single form of AS, AF led with 830 cases, followed by SE with 743, A3 with 644, and A5 with 620. In comparison, AL (96), RI (140), and MX (24) were relatively less frequent. When comparing the distribution of AS patterns across all gene populations with those with a single splicing pattern, the ranking of the proportions of each splicing mode differed markedly (Figure 6B).

### 3.7. Differential Alternative Splicing Events of Developmental Stages and Tissues

Alternative splicing is a pivotal mechanism in gene expression regulation [55]. It plays a crucial role in generating transcriptomic diversity. To systematically investigate differential AS events across developmental stages and tissues in *B. mori*, we conducted comprehensive analyses employing a rigorous statistical criterion in which splicing variants were identified using a FDR ≤ 0.05 as the significance threshold [56].

As detailed in Table 3, significant differences in AS events were observed across distinct developmental stages, with SE consistently representing the most abundant splicing type and RI showing the lowest frequency. The developmental transition between larva_4th_instar and larva_5th_instar exhibited the highest total number of AS events (90,969). Notably, the wandering to moth stage comparison demonstrated both the highest number of alternatively spliced genes (17,616) and the highest count of statistically significant splicing patterns (2712). The most significant concentration of differentially spliced genes (1619) was identified during the transition from larva_2nd_instar to larva_3rd_instar. Although detectable AS events and associated genes were observed between the pre-pupal and pupal stages, none were statistically significant. We analyzed tissue-specific AS events using the aforementioned methodology, with the findings presented in the Supplementary File.

### 3.8. TGS Detected a Higher Number of Alternative Splicing Events

TGS provided new AS information for 4219 silkworm genes, whereas NGS offered such information for 3641 genes. Furthermore, 640 genes with AS information in the reference genome had not been previously reported. We also found that TGS and NGS jointly identified 225 genes, while TGS and REF shared 95 genes. The largest overlap was observed between NGS and REF, with 3135 genes in common. In total, 9898 genes were common across all three methodologies (Figure 6D). In our study of the silkworm genome, TGS uncovered 11,899 novel AS forms, while NGS identified 10,801 AS events. Additionally, 7595 AS events in the REF had not been previously reported. Among the AS events discovered by both TGS and NGS, 214 were common to both; 1039 were shared between TGS and REF, and 12,378 were shared between NGS and REF. In conclusion, 12,388 AS events were shared across all three methodologies (Figure 6E).

We further conducted an in-depth analysis of the AS events discovered by TGS and NGS. The results indicated a significant difference in the types of AS events revealed by these two technologies. Among the total AS events identified in the silkworm genome by TGS, the types of AF, A5, SE, and A3 accounted for a relatively larger proportion, while the MX type accounted for a relatively smaller proportion (Figure 6F). This distribution pattern was consistent with the proportions of AS forms discovered by NGS. An exploration of AS events uniquely identified by TGS or NGS revealed significant differences in the types and proportions of splicing events detected by these two technologies. Specifically, NGS predominantly identified AF, SE, and A3 forms of AS, while the TGS results were characterized by a predominance of A5. Compared to NGS, TGS showed markedly different proportions of AF, SE, and A3 types of AS (Figure 6G).

### 3.9. Xian8 and 9211 Exhibit Significant Differences in Core Alternative Splicing Patterns

A multi-tissue comparative analysis between Xian8 and 9211 revealed highly significant tissue-specific AS patterns. The analysis identified significant AS events in seven key genes, with six genes lacking known functional annotations and only one gene possessing a confirmed functional annotation (Figure 6H and Figure S1). Alternative splicing in these genes exhibited significant heterogeneity both between strains and among tissues. Taking the annotated U1 snRNP C gene as an example, in strain 9211, this gene demonstrated only a single splicing isoform in the midgut tissue, but it exhibited multiple AS patterns in the testis. Notably, in strain Xian8, no detectable AS events were observed in either the fat body or midgut tissues, whereas there was significant splicing diversity in the other three examined tissues (Figure 6H). Comparative analysis of the two silkworm strains (9211 and Xian8) also revealed that the 9211 strain had a higher frequency of AS events across all examined tissues than the corresponding Xian8 strain (Figure 6H).

## 4. Discussion

### 4.1. Significance of HKGs and Tissue-Specific Genes

The RNA atlas of *B. mori* provides a critical foundation for elucidating spatiotemporal gene-expression dynamics, particularly in specialized tissues such as the silk gland. Single-cell RNA sequencing has enabled the construction of a comprehensive cell atlas, revealing 14,972 high-quality cellular and transcriptional profiles across silk gland regions, thereby enhancing our understanding of the spatially specific gene-regulatory mechanisms underlying silk protein synthesis. HKGs, expressed across multiple tissues, exhibited stronger selective constraints compared to TSGs (Figure 3 and Figure 4), reflecting their essential roles in maintaining basal cellular functions (Figures S2 and S3). For example, HKGs involved in lipid metabolism and cellular homeostasis demonstrated conserved expression patterns critical for larval development and nutrient utilization. Conversely, TSGs, such as those encoding silk fibroins or cuticular components, exhibited strict tissue-specificity (e.g., posterior silk gland-specific) and domestication-driven expression divergence, directly influencing silk yield and quality. The integration of RNA atlas data with pan-genomic resources further enables the identification of allelic variants and regulatory networks governing both HKGs and TSGs, offering novel targets for genetic improvement in sericulture.

### 4.2. Regulatory Networks Reveal Specific Gene Expression Modules and Functional Hub Genes

Our findings demonstrate that WGCNA effectively identified gene modules that are strongly associated with specific tissue types and developmental stages in *B. mori*. The hub genes in the midgut-related module were all digestive-enzyme genes, whereas those in the brain-related module were insulin-related genes; these results were supported by high correlations (>0.7). This highlights WGCNA’s significance in constructing robust co-expression networks that reveal functional gene clusters, enabling the prioritization of hub genes involved in specific biological processes such as digestion and metabolic regulation. The reliability of these results is reinforced by the method’s consistency in identifying biologically plausible modules—digestive enzymes align with midgut functions, and insulin genes correspond to brain roles in growth and development.

### 4.3. Panoramic Atlas of Alternative Splicing Based on Third-Generation Sequencing

The construction of a comprehensive AS atlas in the silkworm provides critical insights into its molecular adaptations and functional complexity. Studies have shown that AS plays a pivotal role in sex determination, developmental regulation, and antiviral responses, with sex-specific splicing patterns observed in genes such as Bmdsx [57]. High-resolution pan-genome datasets and single-cell transcriptomic analyses of silk glands further highlight tissue- and stage-specific AS events, linking the diversity of splicing to silk protein synthesis and organogenesis [31,58]. The integration of third-generation sequencing technologies with existing genomic resources enables precise identification of AS events and their evolutionary conservation across Lepidoptera. However, challenges remain in elucidating the regulatory mechanisms underlying AS during domestication and environmental adaptation, particularly for optimizing silk yield and stress resistance. Future studies should leverage multi-omics approaches to analyze AS-mediated post-transcriptional networks and their translational applications in sericulture.

Our research demonstrates that TGS outperformed next-generation sequencing (NGS) in detecting diverse AS isoforms, identifying more complex splicing patterns with only 10 samples compared to nearly 900 NGS transcriptomic datasets. TGS leverages long read lengths, single-molecule resolution, and enhanced structural variant detection to span entire transcript structures and resolve complex splicing junctions that are often missed by short-read technologies, thereby enabling comprehensive characterization of splicing variants [59,60]. TGS exhibits unparalleled sensitivity in identifying low-abundance isoforms and elucidating splicing regulation mediated by structural variants (e.g., genomic rearrangements) [61]. However, the inherent high error rates of nanopore platforms necessitate hybrid correction strategies integrating high-accuracy NGS data to ensure benchmark precision [62]. TGS demonstrated significant potential to elucidate silkworm germplasm characteristics and refine breeding strategies: its long-read capability facilitates high-resolution genome assembly and structural variant detection, thereby revealing rare alleles and complex genomic regions that underpin key economic traits (e.g., silk yield, disease resistance). For example, large-scale pan-genome datasets constructed via TGS have uncovered extensive genetic diversity and strain-specific genomic segments in silkworms, providing a foundation for identifying functional genes and introgression zones between *B. mori* and Bombyx mandarina [22]. Additionally, TGS enables transcriptome-wide analysis of AS events, providing insights into the molecular mechanisms of traits such as BmNPV resistance. Integrated with conventional breeding, TGS not only enhances the accuracy of trait selection but also accelerates the discovery of genetic markers [63], highlighting its transformative role in bridging genomic complexity with practical breeding applications to advance sericulture.

## 5. Conclusions

In conclusion, our study constructed a comprehensive multi-dimensional pan-transcriptome atlas of the silkworm *Bombyx mori*, providing a foundational resource for decoding the spatiotemporal gene expression dynamics and regulatory mechanisms underlying complex biological processes in this model lepidopteran insect. Our work delivers three core biological insights and practical values beyond existing reports: First, we established a high-confidence, standardized panel of housekeeping genes (HKGs) and tissue/developmental stage-specific marker genes. The stably expressed HKGs across the whole life cycle and all tissues provide a universal reference for quantitative gene expression analysis in silkworm, especially for emerging single-cell and spatial transcriptomic studies; while the tissue- and stage-specific genes offer key candidates for dissecting developmental regulation and tissue functional differentiation. Second, our integrated analysis of short- and long-read transcriptomes revealed the widespread, dynamic tissue/strain-specific regulation of alternative splicing (AS) in silkworm, highlighting the critical and previously underappreciated role of post-transcriptional regulation in silkworm development and phenotypic divergence between strains. Third, the full-length transcriptome data we generated supplemented and updated the silkworm genome annotation, and demonstrated the irreplaceable value of long-read sequencing in accurately resolving the complexity of the silkworm transcriptome. This integrated pan-transcriptome resource fills a key gap in lepidopteran functional genomics research. It not only provides a robust reference for gene function studies, regulatory network dissection and multi-omics integration analysis in silkworm and other lepidopteran insects, but also offers abundant molecular markers and candidate targets for molecular marker-assisted breeding, accelerating the cultivation of high-yield and high-quality silkworm varieties.

## Figures and Tables

**Table 1:** Statistics for Tissue-Specific Genes.

Tissue	Gene
antenna	LOC101737574, LOC693104;
brain	LOC101735407, LOC101735848, LOC101740460;
embryo	LOC101737997, LOC101740264, LOC101744972, LOC119630908, novel_gene_63_65869db7;
* embryo_BmE_cells	LOC101746709;
fat_body	LOC101740657, LOC101743298, LOC101744429, LOC101745249, novel_gene_707_65869db7;
head	LOC105841884;
midgut	LOC101735522;
ovary	CPG12_CPG13, CPR91, LOC101741909, LOC10174193, LOC101742474, LOC101742952, LOC10174298, LOC101743125, LOC110386587, LOC119630792;
* ovary_BmN4_cells	Bbx-a7, InR, LOC101736699, LOC101738898, LOC101739472, LOC1017415988, LOC101743234, LOC101744383, LOC101744896, LOC101745044, LOC101745950, LOC119628642, LOC119629137, LOC119629966, LOC119630034, LOC692907, LOC732919, LOC733036, LOC733143, novel_gene_679_65869db7;
silkgland_middle_middle	LOC101745131;
testis	LOC101738468, LOC101738590, LOC101741886,LOC101743393, LOC101745356, LOC101746049, LOC101746875;
thorax	LOC100134921, Rli;

* This tissue classification is a provisional measure for calculation convenience, and remains open to further discussion and refinement.

**Table 2:** Genetic Modules of the weighted gene co-expression network analysis.

Module Colors	Count	Hub Genes	Freq
black	251	5	1.9%
blue	1252	25	9.3%
brown	482	9	3.3%
green	286	5	1.9%
grey	927	18	6.7%
smagenta	100	2	0.7%
pink	219	4	1.5%
purple	81	1	0.4%
red	259	5	1.9%
turquoise	9411	188	69.9%
yellow	389	7	2.6%

**Table 3:** Significant Alternative Splicing (AS) Events Identified From Different Developmental Stages.

Growth1	Growth2	AS Type	Total.AS.num	Total.gene.num	sig.AS.num	sig.gene.num
cell	egg	SE	62,630	8215	51	41
cell	egg	A5SS	2344	1168	8	8
cell	egg	A3SS	2934	1462	9	7
cell	egg	MXE	18,447	4926	517	274
cell	egg	RI	622	411	0	0
egg	larva_2nd_instar	SE	15,241	5121	358	283
egg	larva_2nd_instar	A5SS	1007	676	156	137
egg	larva_2nd_instar	A3SS	1360	877	164	141
egg	larva_2nd_instar	MXE	3018	1623	914	672
egg	larva_2nd_instar	RI	299	232	47	42
larva_2nd_instar	larva_3rd_instar	SE	6095	3238	879	627
larva_2nd_instar	larva_3rd_instar	A5SS	757	558	244	207
larva_2nd_instar	larva_3rd_instar	A3SS	1010	723	312	267
larva_2nd_instar	larva_3rd_instar	MXE	852	490	376	248
larva_2nd_instar	larva_3rd_instar	RI	205	157	72	62
larva_3rd_instar	larva_4th_instar	SE	13,618	5226	449	338
larva_3rd_instar	larva_4th_instar	A5SS	1481	912	55	50
larva_3rd_instar	larva_4th_instar	A3SS	1896	1167	48	40
larva_3rd_instar	larva_4th_instar	MXE	2479	1162	267	136
larva_3rd_instar	larva_4th_instar	RI	442	280	15	15
larva_4th_instar	larva_5th_instar	SE	72,262	9198	190	152
larva_4th_instar	larva_5th_instar	A5SS	4639	1407	38	34
larva_4th_instar	larva_5th_instar	A3SS	5819	1690	44	40
larva_4th_instar	larva_5th_instar	MXE	22,015	5706	2044	954
larva_4th_instar	larva_5th_instar	RI	1395	482	13	10
larva_5th_instar	larva_wandering	SE	69,285	9163	216	168
larva_5th_instar	larva_wandering	A5SS	4300	1392	39	38
larva_5th_instar	larva_wandering	A3SS	5319	1677	40	37
larva_5th_instar	larva_wandering	MXE	21,433	5658	2108	1020
larva_5th_instar	larva_wandering	RI	1317	475	14	13
larva_wandering	moth	SE	46,583	8414	511	386
larva_wandering	moth	A5SS	2862	1322	87	74
larva_wandering	moth	A3SS	3665	1596	75	62
larva_wandering	moth	MXE	12,646	4375	1298	780
larva_wandering	moth	RI	764	449	30	27
moth	pre_pupa	SE	44,276	8327	626	446
moth	pre_pupa	A5SS	2773	1305	86	74
moth	pre_pupa	A3SS	3531	1570	95	79
moth	pre_pupa	MXE	11,648	4148	1309	815
moth	pre_pupa	RI	755	441	35	30
pre_pupa	pupa	SE	37,464	7953	0	0
pre_pupa	pupa	A5SS	2682	1288	0	0
pre_pupa	pupa	A3SS	3280	1567	0	0
pre_pupa	pupa	MXE	9259	3569	0	0
pre_pupa	pupa	RI	763	433	0	0

## Data Availability

TGS data of five distinct tissues from 9211 and Xian8 in this study have been deposited in the NCBI database under project: PRJNA1421089. The data generated and analyzed in this study are available in the EXP832 portal of the web-based Shiny platform (https://wanlinrong.shinyapps.io/EXP832/, accessed on 12 March 2026). The core analytical code for the key analyses in this work has been deposited in a publicly accessible GitHub repository (https://github.com/wanlinrong/EXP832_main_code).

## Supplementary Materials

The following supporting information can be downloaded at: https://www.mdpi.com/article/10.3390/ani16071046/s1, Figure S1: The alternative splicing patterns between xian 8 and 9211; Figure S2: GO enrichment analysis of subclusters 1–4 of Growth_HKGs; Figure S3: GO enrichment analysis of subclusters 1–4 of Tissue_HKGs; Table S1: Go enrichment analysis results of HKGs and SEGs; Table S2: KEGG enrichment analysis results of housekeeping genes; Table S3: Statistics on Growth_HKGs; Table S4: Hub genes in brown and yellow module.

## Abbreviations

AS	Alternative splicing
CV	Coefficient of variation
GTEx	Genotype-Tissue Expression Consortium
HKG	Housekeeping gene
IHEC	International Human Epigenome Consortium
NGS	Next-generation sequencing
PSI	Percent spliced-in
TGS	Third-generation sequencing
TSG	Tissue-specific gene
TPM	Transcripts per million
WGCNA	Weighted gene co-expression network analysis
