# Supplementary material for: Pan-Transcriptome Analyses of Multiple Tissues and Growth Stages Create Expression Atlases for the Silkworm Bombyx mori
Source: Animals (Basel). 2026 Mar 29;16(7):1046. doi: 10.3390/ani16071046 (PMC13072181; doi:10.3390/ani16071046)
Supplement: Supplementary file 1 [file animals-16-01046-s001.zip › Table S1-S4.pdf]

Table S1. Go Enrichment Analysis Results of Housekeeping Genes and Specific Expression Genes.

Type*	GO	Class	Pvalue	Term
Growth	GO:0005230	MF	0.007	extracellular ligand-gated monoatomic ion channel activity
Growth	GO:0004181	MF	0.0076	metallocarboxypeptidase activity
Growth	GO:0003684	MF	0.0117	damaged DNA binding
Growth	GO:0016740	MF	0.0192	transferase activity
Growth	GO:0005198	MF	0.0196	structural molecule activity
Growth	GO:0022857	MF	0.0214	transmembrane transporter activity
Growth	GO:0003924	MF	0.0244	GTPase activity
Growth	GO:0004930	MF	0.0463	G protein-coupled receptor activity
Growth	GO:0008047	MF	0.048	enzyme activator activity
Growth	GO:0006811	BP	0.011	monoatomic ion transport
Growth	GO:0000902	BP	0.019	cell morphogenesis
Growth	GO:0000398	BP	0.022	mRNA splicing, via spliceosome
Growth	GO:0042254	BP	0.036	ribosome biogenesis
Growth	GO:0000288	BP	0.042	nuclear-transcribed mRNA catabolic process, deadenylation-dependent decay
Growth	GO:0031175	BP	0.042	neuron projection development
Growth	GO:0055085	BP	0.049	transmembrane transport
Growth	GO:0007186	BP	0.049	G protein-coupled receptor signaling pathway
Growth	GO:0005761	CC	0.04	mitochondrial ribosome
Growth	GO:0098798	CC	0.041	mitochondrial protein-containing complex
Growth	GO:0005730	CC	0.049	nucleolus
Tissue	GO:0004714	MF	0.035	transmembrane receptor protein tyrosine kinase activity
Tissue	GO:0008234	MF	0.037	cysteine-type peptidase activity
Tissue	GO:0000398	BP	0.031	mRNA splicing, via spliceosome
Tissue	GO:0006749	BP	0.034	glutathione metabolic process
Tissue	GO:0005921	CC	0.046	gap junction

Table S2. KEGG Enrichment Analysis Results of Housekeeping Genes.

Type*	MapID	Pvalue	MapTitle
Growth	map03450	0.0316095248737093	Non-homologous end-joining
Growth	map03018	0.0363504268906581	RNA degradation
Growth_HKG	map05120	0.0423490904341855	Epithelial cell signaling in Helicobacter pylori infection
Tissue_HKG	map04145	0.0109390511741558	Phagosome
Tissue_HKG	map05120	0.011898709427702	Epithelial cell signaling in Helicobacter pylori infection
Tissue_HKG	map03018	0.0150780720805942	RNA degradation
Tissue_HKG	map04130	0.0154605741167197	SNARE interactions in vesicular transport
Tissue_HKG	map04071	0.0331987784416444	Sphingolipid signaling pathway
Tissue_HKG	map03450	0.0334712112942471	Non-homologous end-joining
Tissue_HKG	map04730	0.0434459976692239	Long-term depression
Tissue_HKG	map03015	0.0458514601802114	mRNA surveillance pathway

Table S3 Statistics on Growth-Specific Genes

Growth	Gene
cell	InR,LOC101738772 ,LOC101741598, LOC101746709, LOC119629137, LOC733036, novel_gene_679_65869db7
egg	LOC101740264, LOC101744972, LOC119630908, novel_gene_63_65869db7
larva_5th_instar	LOC101740734,LOC101743393
moth	CPG12_CPG13, LOC101739255,LOC101741235,LOC101741830, LOC101741909,LOC101741931,LOC101742474,LOC101742986, LOC101743125,LOC101743909,LOC101744884,LOC119630728, LOC119631184,novel_gene_525_65869db7

Table S4. Hub Genes in Brown and Yellow Module

Hub_Genes	Kwithin	moduleColor
LOC105842190(trypsin, alkaline C-like)	57.31567869	brown
LOC101738495(trypsin, alkaline A)	57.02946512	brown
Apn2(aminopeptidase N2)	55.97913988	brown
LOC101741476(carboxypeptidase B)	55.64817916	brown
LOC101738003(collagenase)	54.68077941	brown
LOC119631051(serine protease 3-like)	54.44677692	brown
LOC119631137(collagenase-like)	54.35892086	brown
Apn4(aminopeptidase N4)	54.16699931	brown
LOC119628347(homologous with Drosophila Salivary peptide)	54.14740835	brown
LOC119629137(bombyxin-A6)	18.75869417	yellow
Bbx-d1(bombyxin-D1)	18.6846386	yellow
LOC119629139(bombyxin-B5)	18.06760108	yellow
Bbx-a3(bombyxin-A3)	17.55336151	yellow
Bbx-g1(bombyxin-G1)	17.44653969	yellow
Bbx-b5(bombyxin-B5)	17.36488697	yellow
Bbx-b8(bombyxin-B8)	17.27585254	yellow
