# Supplementary figures and images for: Pan-Transcriptome Analyses of Multiple Tissues and Growth Stages Create Expression Atlases for the Silkworm Bombyx mori
Source: Animals (Basel). 2026 Mar 29;16(7):1046. doi: 10.3390/ani16071046 (PMC13072181; doi:10.3390/ani16071046)

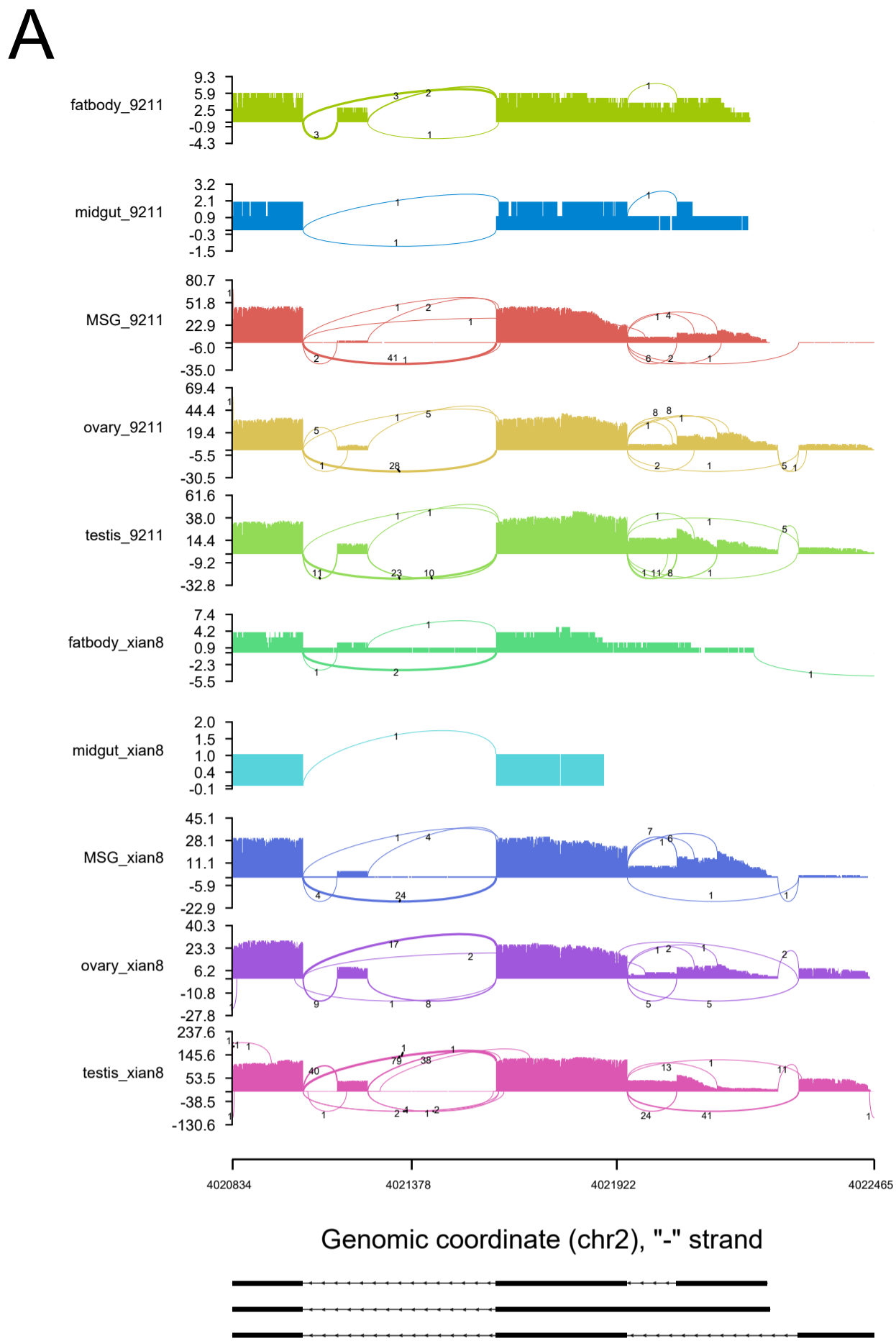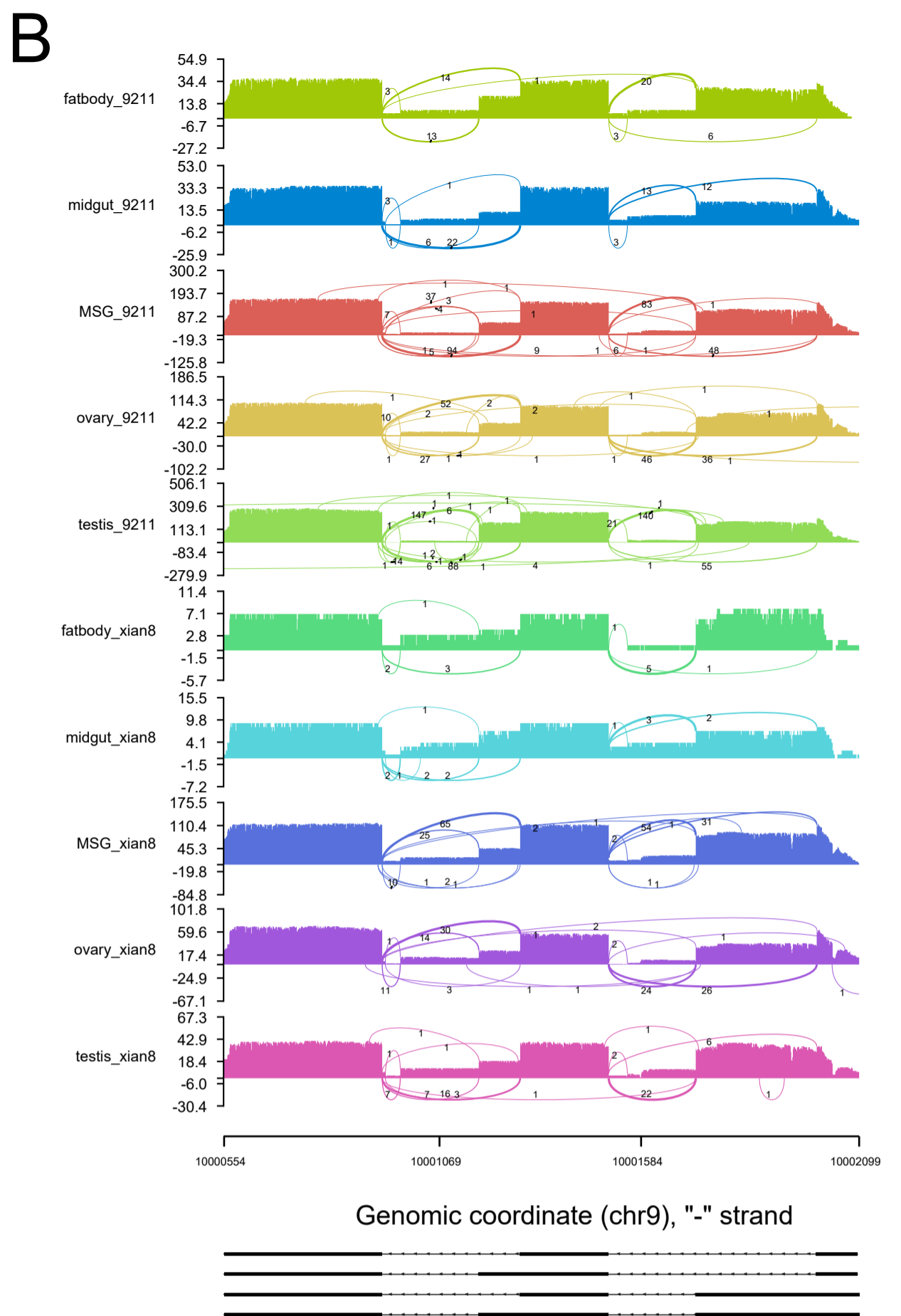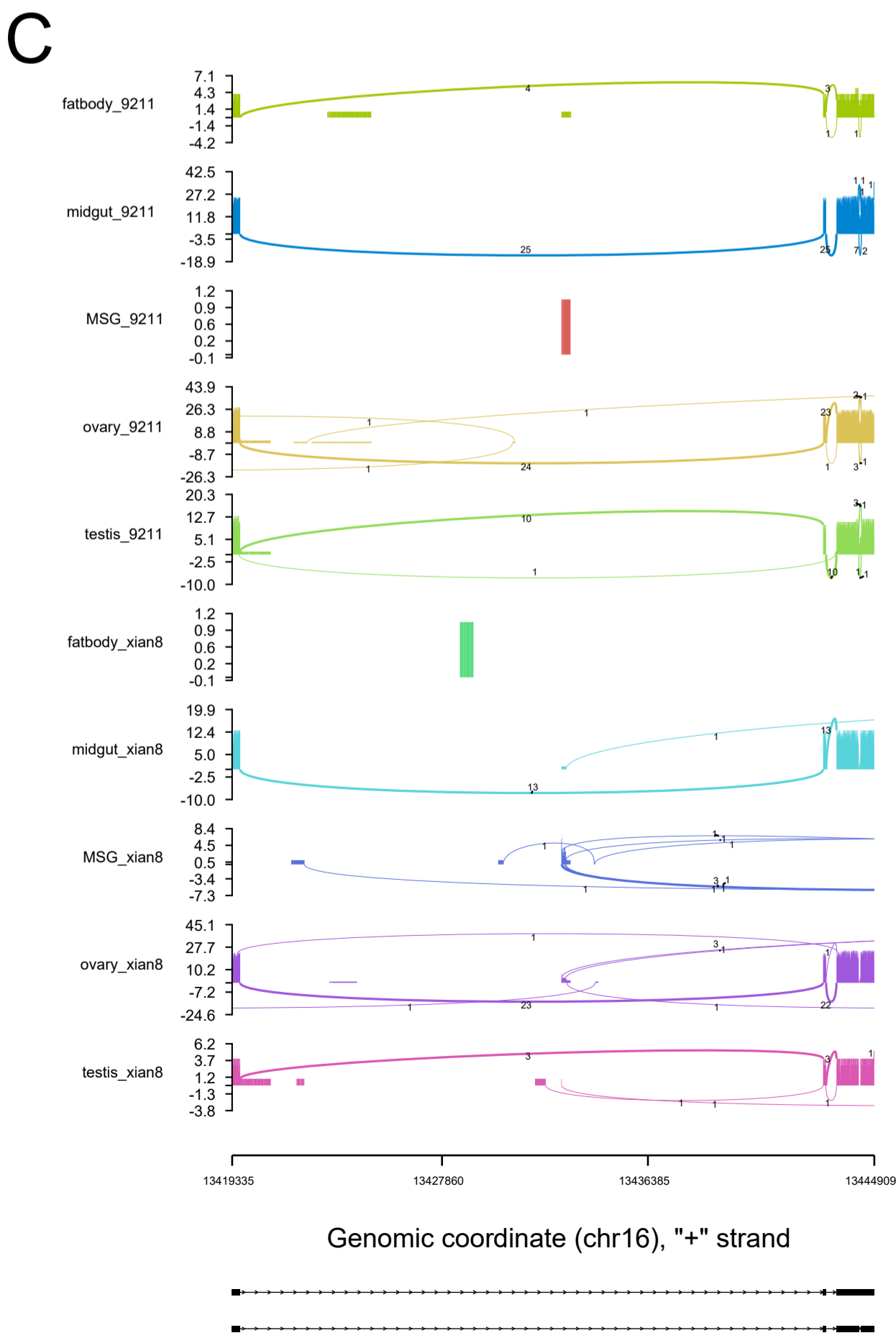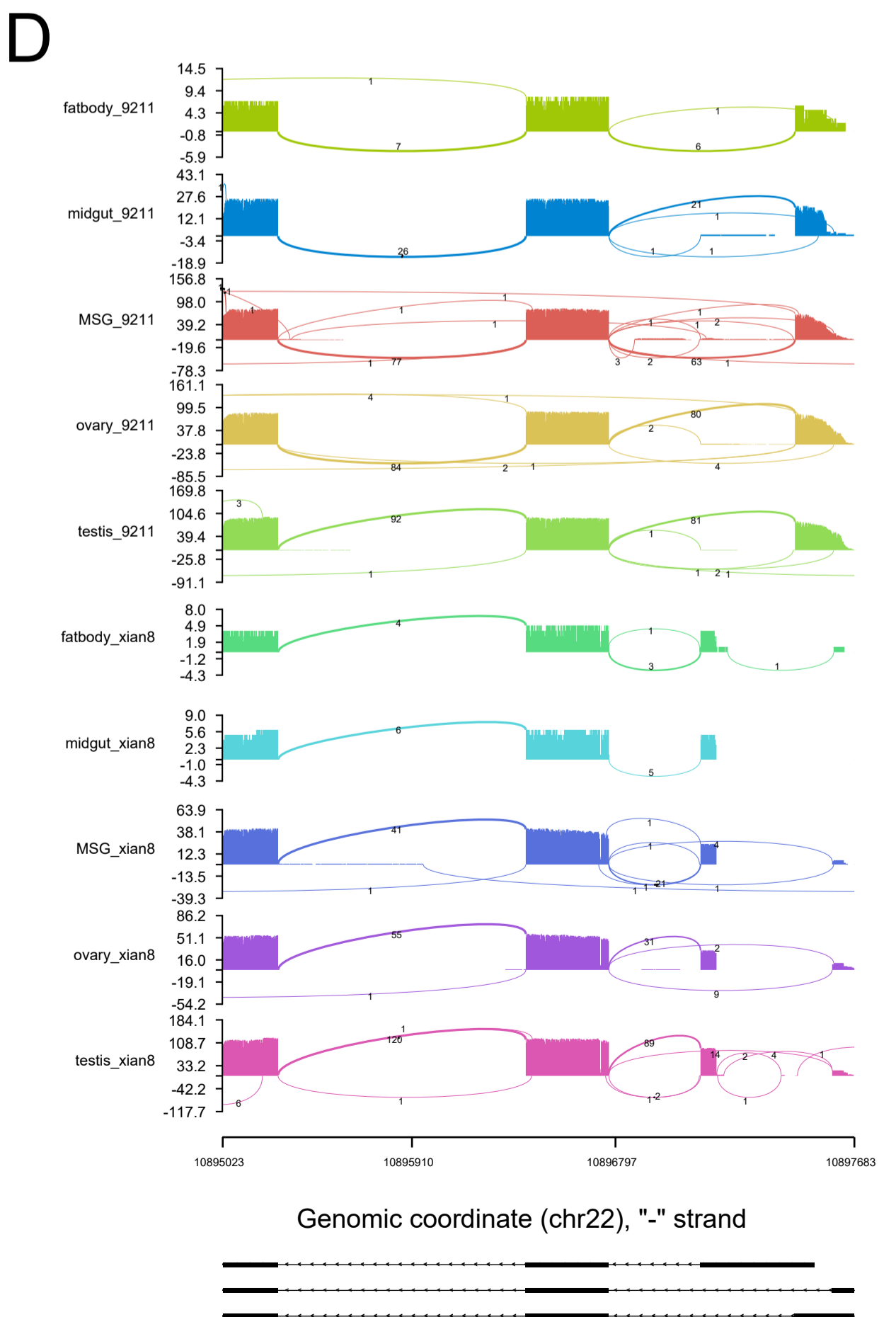

Supplement: Supplementary file 1 [file animals-16-01046-s001.zip › Figure S1.The alternative splicing patterns between xian 8 and 9211.pdf]

Growth\_HKGs

Cluster 1

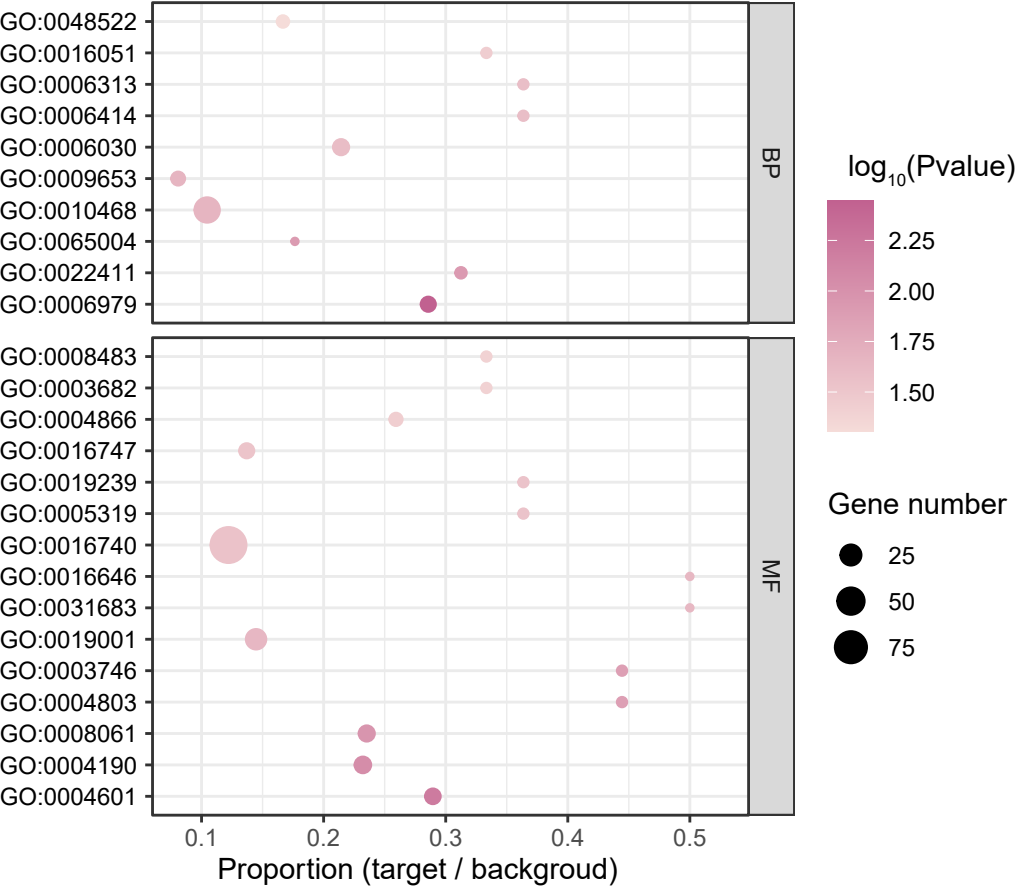

Cluster 2

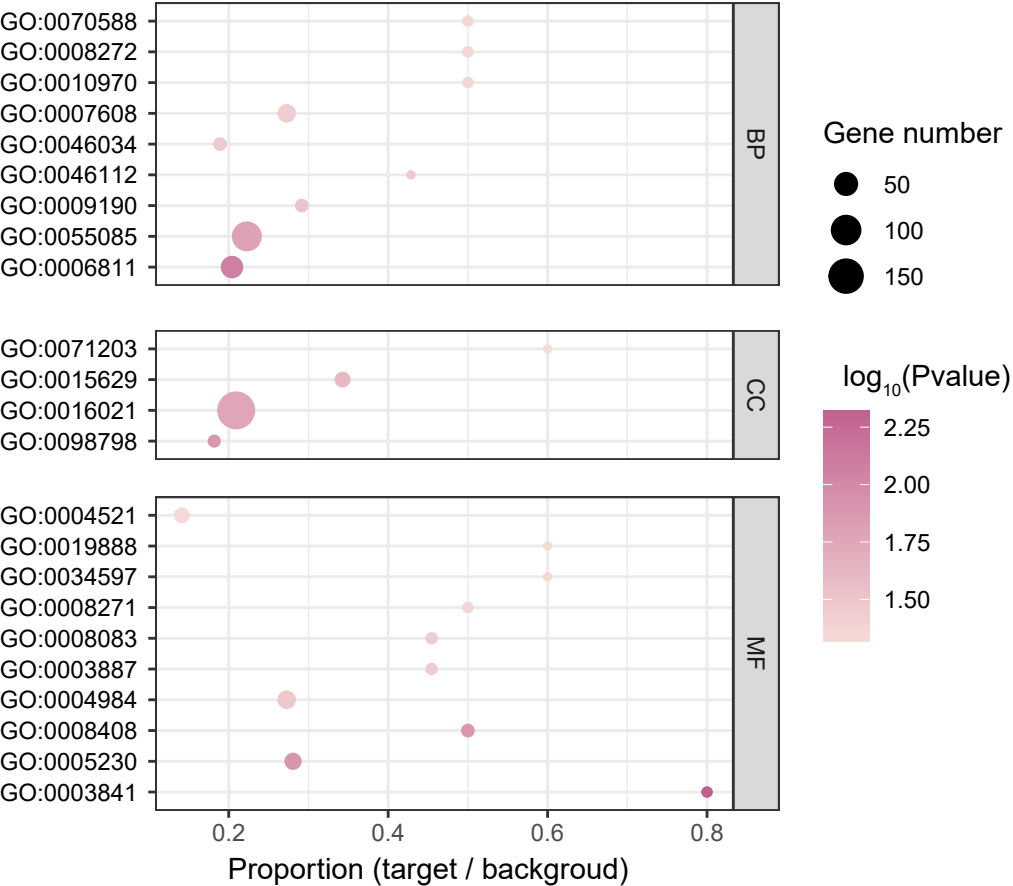

Cluster 3

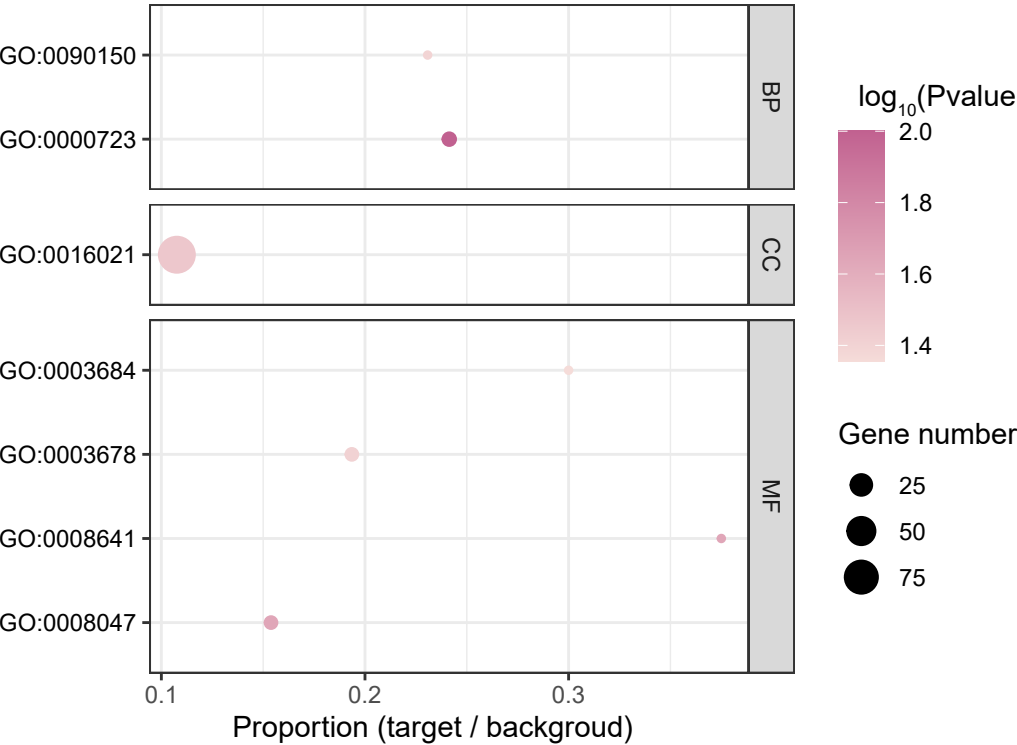

Cluster 4

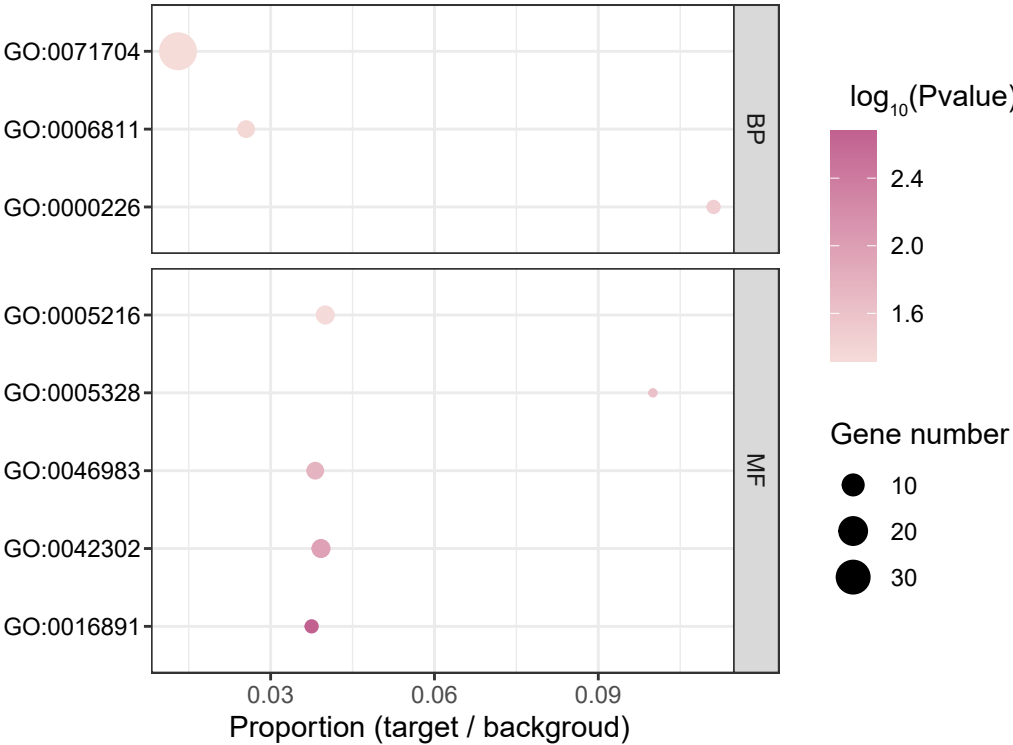

Supplement: Supplementary file 1 [file animals-16-01046-s001.zip › Figure S2.GO enrichment analysis of subclusters 1-4 of Growth_HKGs.pdf]

# Tissue\_HKGs

## Cluster 1

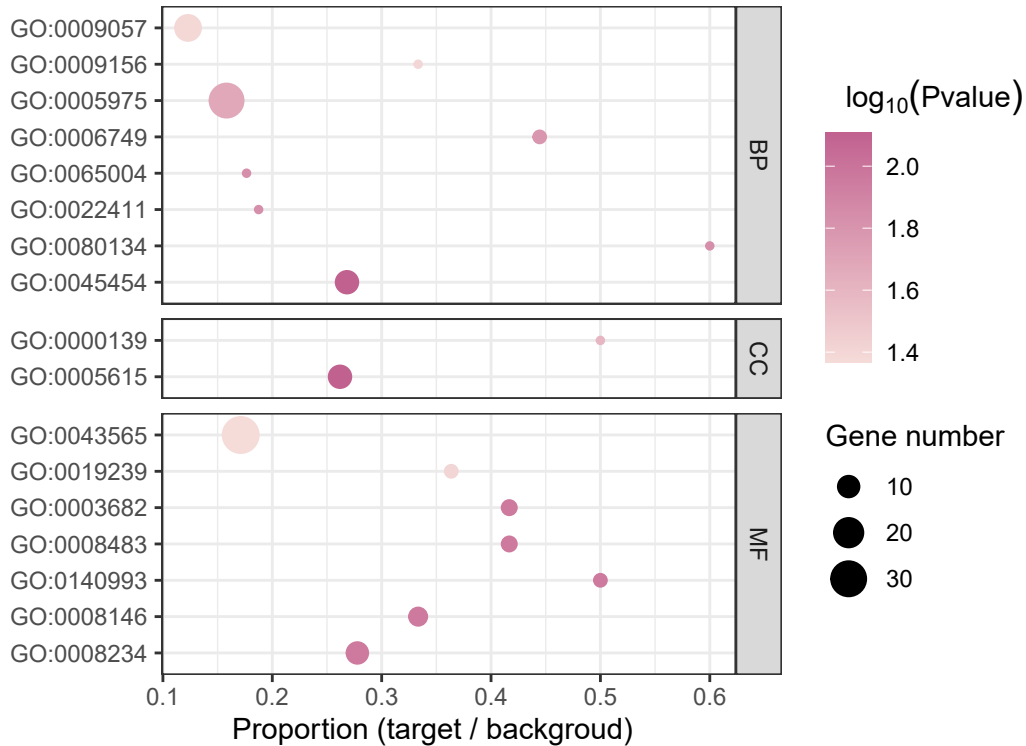

## Cluster 2

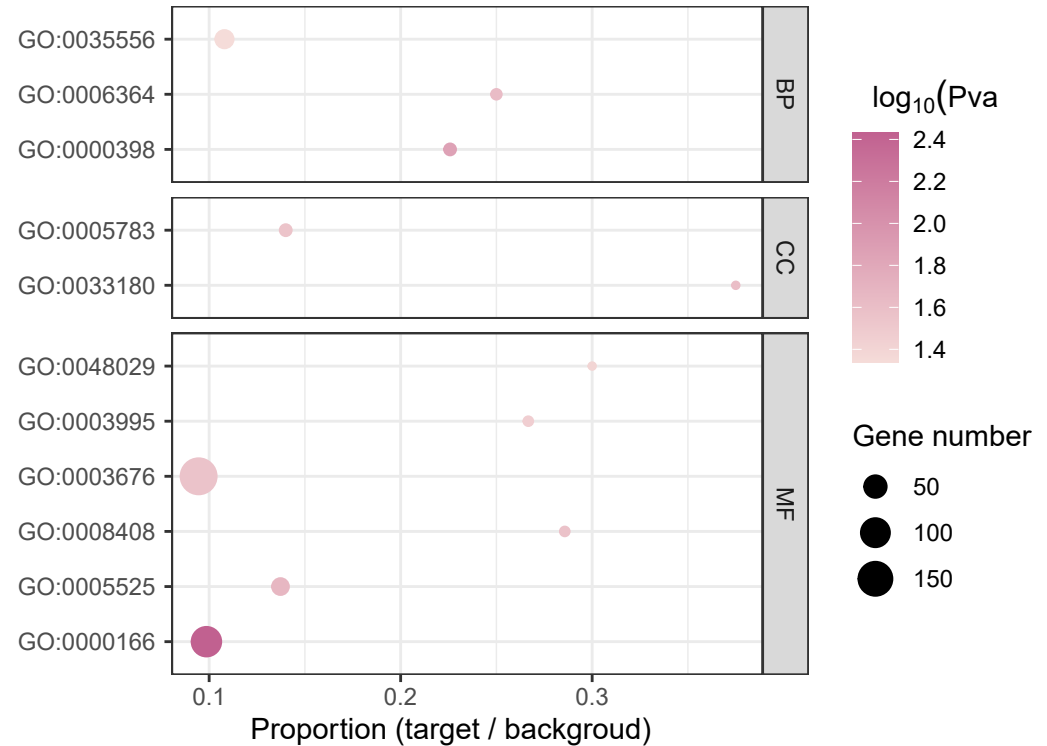

## Cluster 3

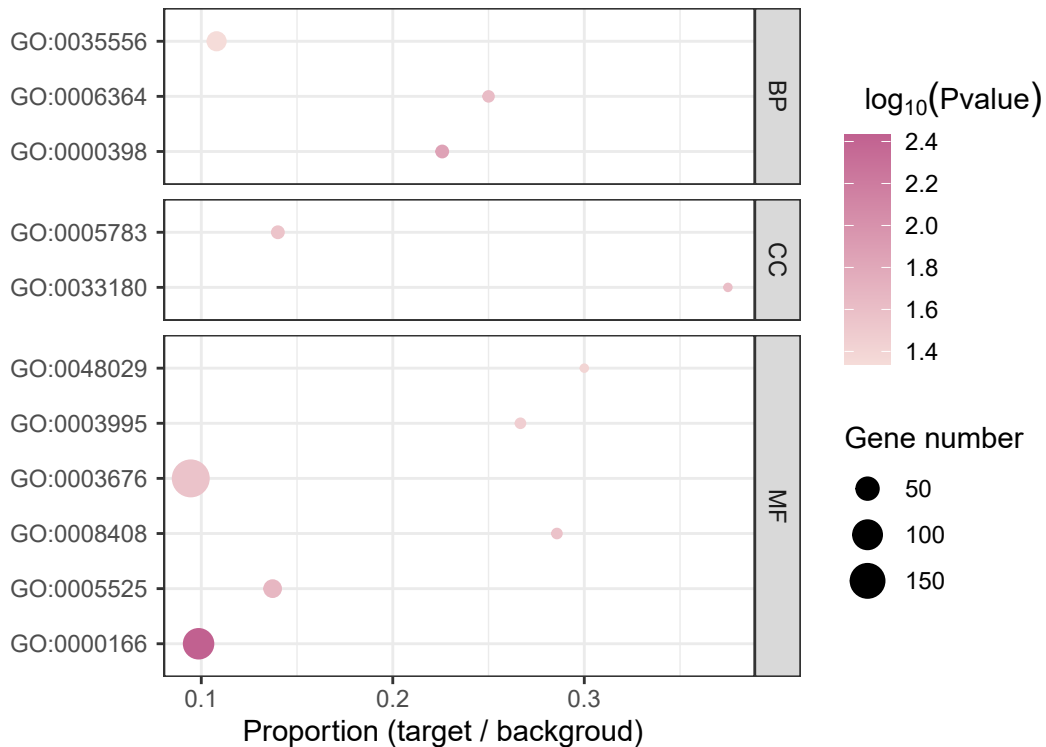

## Cluster 4

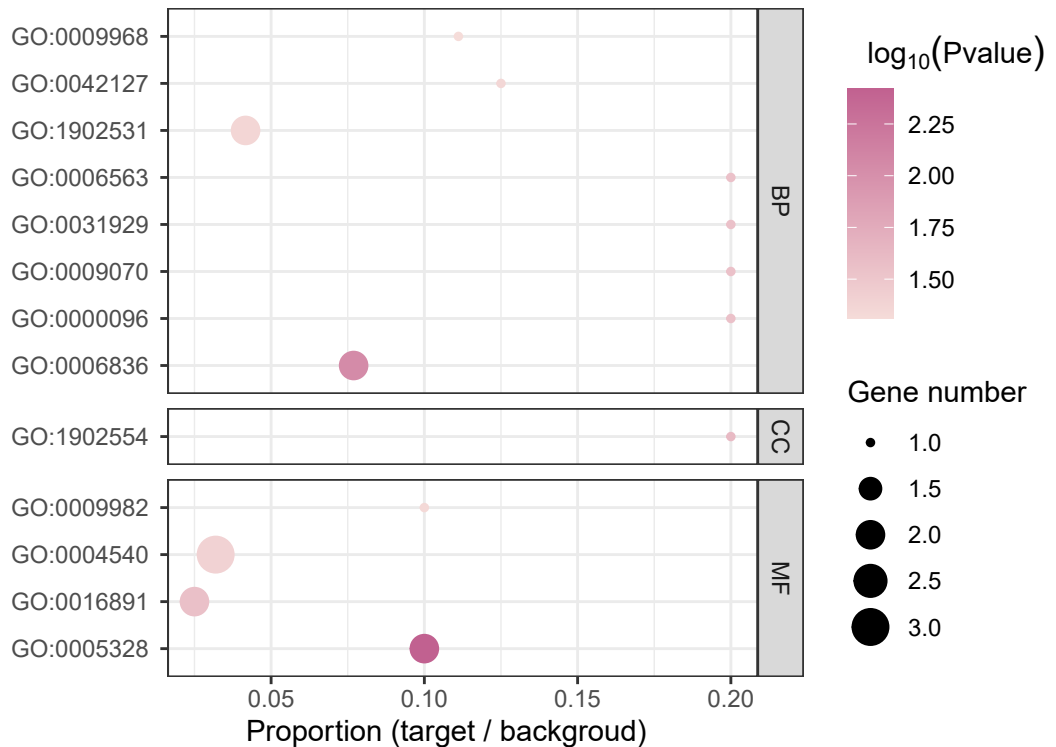

Supplement: Supplementary file 1 [file animals-16-01046-s001.zip › Figure S3.GO enrichment analysis of subclusters 1-4 of Tissue_HKGs.pdf]
